# Parallel simulations for QUAntifying RElaxation magnetic resonance constants (SQUAREMR): an example towards accurate MOLLI T1 measurements

**DOI:** 10.1186/s12968-015-0206-1

**Published:** 2015-11-26

**Authors:** Christos G. Xanthis, Sebastian Bidhult, George Kantasis, Einar Heiberg, Håkan Arheden, Anthony H. Aletras

**Affiliations:** Cardiac MR Group, Department of Clinical Physiology and Nuclear Medicine, Skåne University Hospital Lund, Lund University, Lund, Sweden; Department of Computer Science and Biomedical Informatics, University of Thessaly, Lamia, Greece; Laboratory of Computing and Medical Informatics, School of Medicine, Faculty of Health Sciences, Aristotle University of Thessaloniki, Thessaloniki, Greece; Department of Biomedical Engineering, Faculty of Engineering, Lund University, Lund, Sweden; Centre of Mathematical Sciences, Faculty of Engineering, Lund University, Lund, Sweden

**Keywords:** Magnetic resonance imaging, Mapping, MOLLI, Simulations, Relaxometry

## Abstract

**Background:**

T1 mapping is widely used today in CMR, however, it underestimates true T1 values and its measurement error is influenced by several acquisition parameters. The purpose of this study was the extraction of accurate T1 data through the utilization of comprehensive, parallel Simulations for QUAntifying RElaxation Magnetic Resonance constants (SQUAREMR) of the MOLLI pulse sequence on a large population of spins with physiologically relevant tissue relaxation constants.

**Methods:**

A CMR protocol consisting of different MOLLI schemes was performed on phantoms and healthy human volunteers. For every MOLLI experiment, the identical pulse sequence was simulated for a large range of physiological combinations of relaxation constants, resulting in a database of all possible outcomes. The unknown relaxation constants were then determined by finding the simulated signals in the database that produced the least squared difference to the measured signal intensities.

**Results:**

SQUAREMR demonstrated improvement of accuracy in phantom studies and consistent mean T1 values and consistent variance across the different MOLLI schemes in humans. This was true even for tissues with long T1s and MOLLI schemes with no pause between modified-Look-Locker experiments.

**Conclusions:**

SQUAREMR enables quantification of T1 data obtained by existing clinical pulse sequences. SQUAREMR allows for correction of quantitative CMR data that have already been acquired whereas it is expected that SQUAREMR may improve data consistency and advance quantitative MR across imaging centers, vendors and experimental configurations. While this study is focused on a MOLLI-based T1-mapping technique, it could however be extended in other types of quantitative MRI throughout the body.

## Background

In the field of cardiovascular magnetic resonance (CMR) T1 mapping, quantitative measures of myocardial and blood T1 enabled the calculation of important myocardial biomarkers, such as extracellular volume (ECV) fraction and native T1 in the myocardium [1, 2]. Recent advances in T1 mapping include various new techniques such as Modified Look-Locker inversion recovery (MOLLI), Saturation recovery single-shot acquisition (SASHA), AIR, SAPPHIRE and Shortened MOLLI (ShMOLLI) [3–7]. Moreover, there have been significant efforts towards understanding how accuracy, precision and reproducibility of these methods are affected by acquisition and post-processing parameters [8–11].

While T1 mapping has the potential to improve patient diagnosis, the challenge remains in acquiring reliable data in terms of accuracy and precision. Different T1 mapping methods and parameter sets should yield similar results for a specific tissue type. However, the complex nature of the underlying physics involved and the multitude of parameters that affect image acquisition and post-processing do not allow for consistent reference T1 values of normal myocardium and blood across all methods. Examples of this inconsistency include recent studies on cardiac T1 mapping, which have reported different ranges of T1 values for normal myocardium and blood depending on the methods being used [3, 6, 11–13].

More recently, a single vendor, multicenter clinical study [14] demonstrated reproducibility of myocardial T1 values and provided data from healthy myocardium. Interestingly, the reported T1 values in the literature covers a large range depending on the methods [8]. The T1 mapping technique (MOLLI) and the acquisition parameter set used in the aforementioned multicenter study are listed [14] as limiting factors in terms of accuracy when compared to other setups (different T1 mapping techniques, acquisition scheme, flip angle etc.) [8, 10].

The development of new CMR techniques for obtaining quantitative information usually involves a two-step process: acquisition of images from the MRI scanner with a custom designed pulse sequence and post-processing of the acquired images through a data fitting procedure with closed form equations. Recently, a novel approach to collect and process images for extracting quantitative data from MRI experiments was proposed [15]. This new approach relies on making pseudorandom measurements and comparing the rapidly acquired data against a large dictionary of Bloch simulations. When the matching dictionary entry is found then the tissue constants are known since each of the entries was created based on specific tissue constants. This approach is new in quantitative MRI but requires a uniquely designed pulse sequence with a continuous variation of the acquisition parameters throughout the data collection.

In the past, CMR simulations have been used within a limited scope. Simulations have been used to identify the effect of various acquisition parameters on measurement accuracy and precision [10, 16] and to produce T1-maps through an inverse problem solving fitting procedure [17]. However, in order for the simulator to be executed within a reasonable amount of time, several assumptions and compromises on pulse sequence design had to be made (such as the addition of a crusher to reduce T2-dependency). For the same reason some realistic aspects of the experiment (such as excitation slice profile) had not been incorporated.

In this study, we propose Simulations for QUAntifying RElaxation Magnetic Resonance constants (SQUAREMR), a new method for extracting quantitative tissue MR data from clinical pulse sequences with the aid of comprehensive, parallel MRI simulations of the Bloch equations. The specific aim was to utilize realistic simulations of MOLLI on a large population of spins so as to compute all possible outcomes of this pulse sequence for a range of physiologically relevant tissue relaxation constants. We hypothesized that quantitative CMR acquired with MOLLI can be improved by comparing the signals acquired from the MRI scanner to the entire pool of possible outcomes that are produced by these simulations for different tissue types. While this study is focused on a MOLLI-based T1 mapping technique, it could however be extended in other types of quantitative MRI throughout the body.

## Methods

### MOLLI theory and pitfalls

**Fig. 1:**
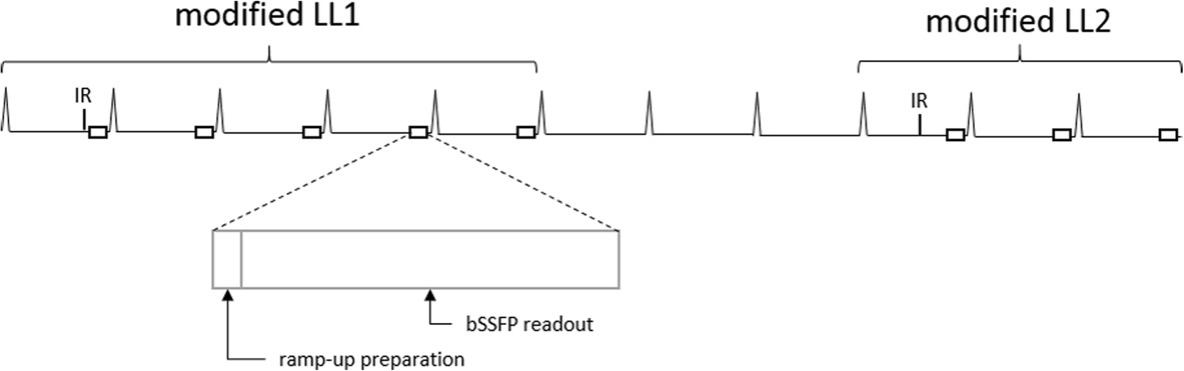
MOLLI acquisition scheme 5(3p)3. Illustration of a MOLLI pulse sequence scheme consisting of two successive ECG-triggered modified-Look-Locker experiments (modified LL1 and modified LL2) with five and three single-shot readouts respectively. A pause of three heart beats has been retained between the two modified-Look-Locker experiments. Each single-shot acquisition consists of a ramp up preparation followed by a balanced steady-state free precession (bSSFP) readout

Figure 1 demonstrates a basic MOLLI pulse sequence scheme [5], where two inversion-recovery-prepared modified-Look-Locker experiments are separated by a pause, which is usually defined in terms of a number of heart cycles. In short, each modified-Look-Locker experiment consists of ECG-triggered single-shot acquisitions performed at end-diastole of consecutive heart beats. Each single-shot acquisition consists of a ramp up preparation (a startup sequence where the flip angle is increased linearly) followed by a balanced steady-state free precession (bSSFP) readout. For every modified-Look-Locker experiment, the effective inversion times (TIs) of the single-shot images are defined by the time measured between the end of the inversion recovery (IR) radiofrequency pulse and the center of k-space of each bSSFP readout within the same modified-Look-Locker experiment. At the end of the MOLLI experiment, the acquired images undergo exponential fitting on a pixel-by-pixel basis in order to estimate Τ1.

In MOLLI T1 mapping, the bSSFP readouts that follow the IR pulse perturb the relaxation process itself and do not allow for an ideal exponential recovery that is based solely on the equilibrium magnetization (M0) and the relaxation constant T1. As a result, the true recovery follows an apparent relaxation constant T1* which is always shorter than the true relaxation constant T1. MOLLI reconstruction compensates for this apparent relaxation by means of a 3-parameter exponential signal model and the “Look-Locker” correction factor [5]. However, the “Look-Locker” correction factor has been derived based on a Fast Low Angle Shot (FLASH) readout [18] and is used for the bSSFP readout since a simple closed form expression does not exist for the actual MOLLI pulse sequence. Although it has been shown that the FLASH-based correction factor is reasonably effective under specific conditions [10], it does lead to inaccuracies in T1 maps.

### SQUAREMR overview

The basic concept of this study is based on the premise that the signals obtained from the simulation of a clinical pulse sequence on simulated tissue with specific relaxation constants (T1, T2) would be identical to the measured signals acquired from the MRI scanner by applying the same pulse sequence on real tissue with the same relaxation constants (T1, T2). Therefore, for the same pulse sequence, obtaining identical simulated and measured signals would ideally indicate that the tissue constants were identical in both the simulation and the MRI scanner. Using a closed form expression of the clinical pulse sequence was not necessary but instead the identical pulse sequence was simulated and was applied on a computer model of spins. The solutions of the Bloch equations provided the temporal evolution of each spin magnetization vector under the influence of the RF pulses and magnetic field gradients of the pulse sequence.

**Fig. 2:**
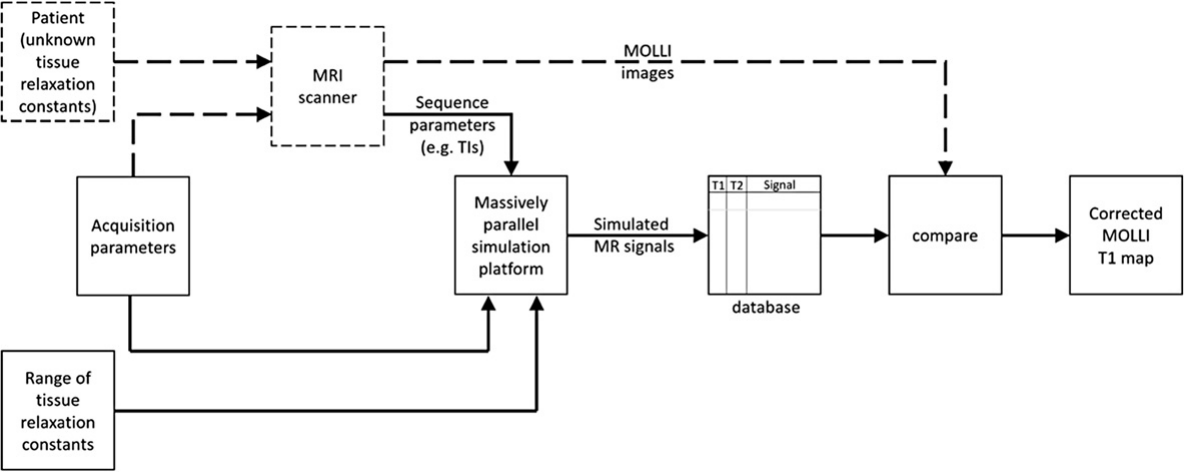
Block diagram of SQUAREMR. The dashed lines demonstrate the conventional MOLLI workflow whereas solid lines illustrate the workflow steps introduced by SQUAREMR. (HR = heart rate)

A basic block diagram of SQUAREMR is shown in Fig. 2. In a conventional manner (dashed lines), the patient is scanned in an MRI scanner by applying a user-selected acquisition parameter set. Then, the MRI signals, acquired from the MRI scanner, are processed in order to quantify tissue relaxation constants. With SQUAREMR, several steps were added to this workflow (solid lines). The identical acquisition parameter set was used by a parallel simulator to process the entire range of physiological tissue relaxation constants. The noise-free simulated signal was sampled at the exact inversion times (TIs) used in the MRI scanner with MOLLI. Then, a custom database of simulated signals was constructed so as to link each recorded simulated signal to a single pair of T1 and T2 values. Then, for the MOLLI images acquired by the scanner, on a pixel-by-pixel basis, the relaxation times T1 and T2 were estimated by finding the simulated signal in the database that produced the least squared difference to the MOLLI MRI signal within a pixel. In other words, when this least squared difference was identified, the related database entry returned the tissue relaxation constants.

### Acquisition parameters

The acquisition parameters described the MOLLI pulse sequence. These included the following parameters: number of inversion-recovery-prepared modified-Look-Locker experiments, number of ECG-triggered single-shot acquisitions per modified-Look-Locker experiment, number of pausing heart cycles between modified-Look-Locker experiments, IR RF pulse duration and shape, TR and TE durations, bSSFP readout RF pulse duration and shape, slice thickness, acquisition scheme, Field-Of-View (FOV), matrix size, SENSE factor, receiver bandwidth (rBW) and number of startup TRs in the linear flip angle ramp.

In MOLLI, the TIs for a number of the single-shot images depend on the actual heart rate and cannot be known before the pulse sequence execution. Therefore, the simulations were performed after image acquisition.

### Parallel simulation platform

A comprehensive MR physics simulator, MRISIMUL, was used [19, 20]. Compared to other previously developed MR simulators, MRISIMUL is neither an image-based simulator that utilizes T1, T2 and PD maps in combination with equations that describe the image intensity [21] nor a kspace-based simulator that utilizes the k-space formalism [22]. MRISIMUL is a simulation platform based on discrete-event Bloch equations applied on anatomical models of spins that incorporates realistic aspects of the MR experiment, makes no assumptions or simplifications for simulating the underlying MR physics and exploits parallel computing based on Graphics Processing Units (GPUs) for high computational performance. The computationally demanding core services (kernel) of MRISIMUL were developed in CUDA-C (NVIDIA, Santa Clara, CA) and distributed in parallel within the graphic processing units (GPUs) whereas the simulation wrapper was developed in MATLAB (The Mathworks Inc., Natick, MA, USA).

For each MOLLI experiment, the identical pulse sequence was simulated on a population of spins for a large range of physiological combinations of T1 and T2. T1 and T2 values of 200–1900 msec and 20–400 msec respectively were simulated with a step of 1 msec. Simulations were not performed for combinations where T2 > T1. To explore faster SQUAREMR execution times, a step of 5 msec was also used. The ranges of T1 and T2 values were chosen based on physiological myocardial and blood values found in the literature [10, 14] with an expanded range based on the characteristics of each experiment. For example, since no gadolinium was administered, very short T1 values below 200 msec were not considered. For each one of the simulations, the MOLLI pulse sequence was applied on a spin with unique characteristics (T1, T2 and position along the slice direction). The simulated MOLLI pulse sequence was based on the pulse sequence run on the MRI scanner. The receiver bandwidth (rBW) of the MRI scanner also defined the temporal step Δt of the simulated pulse sequence. A total of approximately 75,000 to 150,000 time steps were computed for each simulation. The Bloch simulation temporal resolution was 10 μsec and 5 μsec respectively. The bSSFP condition was retained throughout the simulated MOLLI pulse sequence, whereas software crushers [19] were utilized before and after the IR pulse. Also, in order for realistic slice profiles to be incorporated in the simulations, approximately 20 to 100 spins were simulated across the slice thickness. A total of approximately 532,000 to 63,400,000 simulations of the entire imaging pulse sequence were performed. The resulting database consisted of a total of approximately 25,000 to 628,000 entries respectively.

Simulations were performed on a single-node system consisting of a server style computer of 2 hexa-core (Intel E5-2630, 2.30 GHz) processors, 32 GB RAM and four Tesla C2075 GPU cards. Each Tesla C2075 graphics card utilized a total dedicated memory of 6 GB GDDR5 and a total of 448 stream processors.

### SQUAREMR performance

To investigate SQUAREMR performance, two sets of pre-Gd MOLLI experiments were performed. For a given experiment, the total SQUAREMR execution time could be reduced by decreasing either the total number of spins within the spin model or the total number of pulse sequence time steps or the T1 map size or a combination of them.

The first set of MOLLI experiments was performed with a simulation temporal step of 10 μsec and an acquisition matrix size of 128x128 whereas the second set of experiments was performed with a simulation temporal step of 5 μsec and an acquisition matrix size of 320x320. To further investigate how spin model size relates to SQUAREMR performance, two different test cases were considered for each set of experiments. For the first test case, a short T1 and T2 sampling step of 1 msec was combined with a large number of 101 spins along slice thickness resulting in a total of approximately 533,000 database entries. For the second test case, a longer T1 and T2 sampling step of 5 msec was combined with a small number of 21 spins along slice thickness resulting in a total of approximately 21,500 database entries. For both test cases, T1 and T2 values of 600–2000 msec and 20–400 msec respectively were simulated.

### Phantom setup

Two phantoms of six “tissues”, each with its own T1 and T2 values, were used in this study. The phantoms were prepared with varying concentrations of CuSO_4_ and Agar [23] in order to obtain specific combinations of T1 and T2 values. Agarose powder was weighted and dissolved in distilled water and the proper amount of a 10 mM CuSO_4_ solution was added. The mixture was heated, poured into containers (one per “tissue”) and was left to reach room temperature.

The first phantom consisted of six “tissues” (plastic bottles of 500 ml) with target T1 and T2 values close to real cardiac tissues relaxation constants found in the literature, both with and without gadolinium contrast agent present. The “tissues” used in this set had T1s and T2s of pre-contrast normal myocardium [11, 24], pre-contrast blood [10, 25], pre-contrast edematous myocardium [26], pre-contrast infarcted myocardium [5, 24], post-contrast normal myocardium (2–3 min after gadolinium contrast administration) and post-contrast normal myocardium (13–15 min after contrast administration) [27, 28]. The second phantom set consisted of 6 “tissues” (Eurospin II Test System, Livingston, UK) with T2 values close to T2 of normal myocardium [24] and T1 values covering the range from 200 msec to 1600 msec.

To study the SQUAREMR T2 estimates obtained with the MOLLI sequence a third set of 10 phantoms of varying combinations of T1 and T2 values was scanned with the clinically relevant 5(3p)3 scheme only. The target T1 and T2 values were chosen so as to cover the following four combinations: short T1 and short T2 values; short T1 and long T2 values; long T1 and short T2 values; long T1 and long T2 values.

Relaxation constants reference standard values were measured on a 1.5 T Philips Achieva systems (Philips Healthcare, Best, Netherlands). For T1 measurements Saturation Recovery was used (Tsat = 0.01–15 sec, TR = 10 sec) because it allowed for visual evaluation of the effectiveness of the saturation pulse at the shortest saturation time i.e. for long T1s, the remaining signal was within or uniformly close to the noise floor. For T2 measurements T2p-SSFP was used for faster data acquisition since it has been validated in the past as a reference standard against slow spin-echo experiments [29].

### Healthy volunteer population

Twelve (12) healthy volunteers with no medical history (12 men, age 34 ± 12 years) were studied. Eight out of twelve were studied with the clinically relevant 5(3p)3 scheme only. The study was approved by the local ethics committee and all subjects provided written consent (The regional ethics committee, Lund, Sweden. Ethics application number: 541/2004).

### CMR protocol

CMR studies were performed on a 1.5 T Philips Achieva scanner (Philips Healthcare, Best, Netherlands) equipped with a 32-channel receiver coil and advanced research packages for cardiac applications. The CMR protocol included a series of MOLLI pulse sequences with different acquisition schemes:

1. 5(3p)3 (clinical MOLLI pulse sequence for pre-contrast myocardial T1 mapping) [9, 10]
2. 4(1p)3(1p)2 (clinical MOLLI pulse sequence for post-contrast myocardial T1 mapping) [10]
3. 5(0p)3 (custom MOLLI pulse sequence for shorter myocardial T1 mapping acquisition)

where p stands for heartbeats pauses between modified-Look-Locker experiments (Fig. 1).

All MOLLI pulse sequences shared the following parameters: the IR pulse was a hyperbolic secant adiabatic pulse [30] with 4.74 msec duration, whereas the bSSFP readout used a 490 μsec sinc shaped RF pulse with 6 mm slice thickness and 35° excitation flip angle, rBW 200 kHz (1612.9 Hz/pixel), field-of-view (FOV) 272 mm×272 mm, acquisition matrix 124×124, linear k-space trajectory and SENSE acceleration factor of 2 (actual number of phase encoding steps was 65). A linear ramp up preparation of 10 pulses was used to reach steady state prior to the bSSFP readout.

In phantom studies TR was set to 2.54 msec and TE to 1.27 msec. MOLLI schemes 1 and three used initial TIs equal to 114 msec and 350 msec whereas MOLLI scheme 2 used initial TIs equal to 114 msec, 232 msec and 350 msec (initial TI increment of 118 msec). For phantoms, a 6-channel head coil was used along with a simulated ECG (60 beats per minute). A coronal single slice was acquired for the myocardial phantom whereas an axial single slice was acquired for the Eurospin II phantom.

In healthy volunteer studies the TR was 3 msec and the TE 1.5 msec. MOLLI schemes 1 and 3 used initial TIs of 134 msec and 350 msec whereas MOLLI scheme 2 used initial TIs of 134 msec, 242 msec and 350 msec (initial TI increment of 108 msec). The CMR protocol was applied in a single mid-ventricular short axis slice with a 32-channel receiver coil.

The term “initial TI” was defined as the first TI measured between the end of the adiabatic inversion pulse and the center of k-space of the first single-shot bSSFP readout that followed within the same modified-Look-Locker experiment. The TIs between the inversion pulse and the center of the other bSSFP readouts within the same modified-Look-Locker experiment were determined by the initial TI and the duration of the cardiac cycles preceding each readout.

### Image analysis

**Fig. 3:**
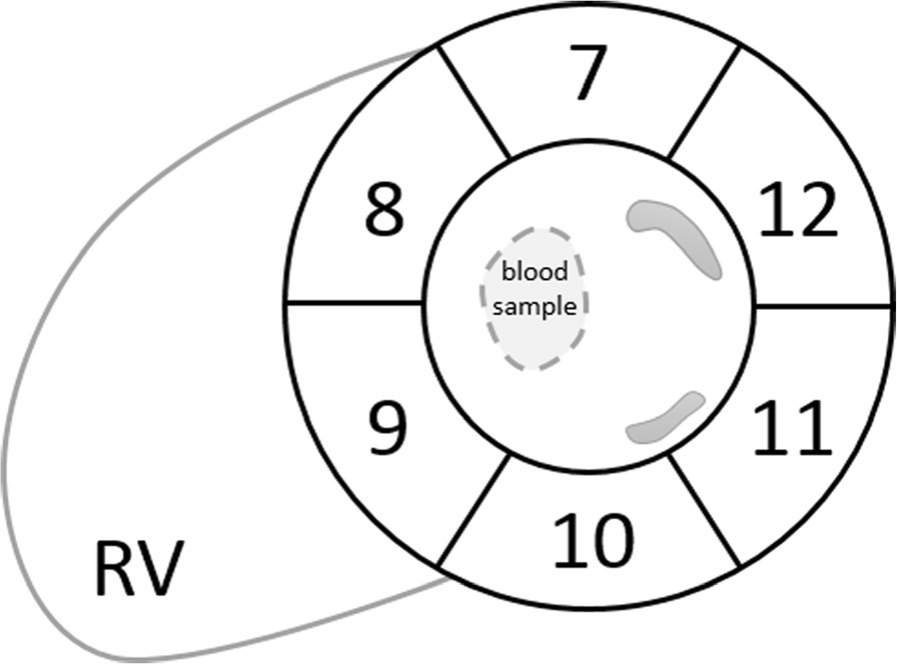
Myocardial segmentation and blood region of interest in a mid-ventricular short-axis slice. Display of the six myocardial segments [32] and the region of interest within the blood pool from which blood relaxation constants were measured (dashed area)

Parameter mapping with SQUAREMR was performed using the GPU-framework of MATLAB (The Mathworks Inc., Natick, MA, USA) on a single GPU whereas MOLLI T1 values were measured from the MOLLI magnitude images using conventional MOLLI post-processing (i.e. with a FLASH-based Look Locker correction factor [5, 18]) with a 3-parameter fit [5, 10]. In-vivo myocardial segmentation was implemented manually [31]. For phantom studies, relaxation constants were measured by placing a rectangular region of interest (ROI) in the center of each phantom and estimated T1 values were reported. For the third set of phantoms, which was intended for studying the T2 estimates, both T1 and T2 values were reported. In healthy volunteer studies, left-ventricular (LV) myocardium was segmented in 6 areas in the mid-ventricular short axis (SAX) slice [32] and relaxation times were measured for each segment separately but also for the entire LV myocardium. Blood T1 and T2 values were measured from an ROI placed within the LV blood pool (Fig. 3). Myocardium and blood ROIs were drawn so as to avoid signal contamination from adjacent tissues. All values in this study are reported as mean ± standard deviation (SD). For phantom studies, modified Bland-Altman plots [33] were used to demonstrate the agreement of the two methods (SQUAREMR and conventional MOLLI post-processing) with the reference standards.

### Statistics

Comparisons were performed with student’s two tailed *t*-test for paired data.

## Results

### Phantom studies

The reference T1 and T2 values of the “tissues” in the first phantom are shown in Table 1. The reference T1 relaxation times of the “tissues” in the second phantom ranged from 212 msec to 1522 msec whereas the reference T2 relaxation times was kept close to that of normal myocardium (52 ± 6 msec).

**Table 1:** Reference T1 and T2 values of the “tissues” in the myocardium phantom

Tissue type	T1 [ms]	T1 StDev [ms]	T2 [ms]	T2 StDev [ms]	CuSO4 [g/L]	Agarose [g/L]
Normal myocardium (pre-contrast)	1048	12	50	2	0,12	17.80
Blood (pre-contrast)	1570	20	196	11	0,07	4.00
Edema (pre-contrast)	1249	15	62	3	0,09	14.67
Infarct (pre-contrast)	1361	21	64	3	0,07	14.51
Normal myocardium (post-contrast 2–3 min)	344	6	52	4	0,2	20.00
Normal myocardium (post-contrast 13–15 min)	413	12	50	3	0,5	17.51

T1 and T2 values were measured with Saturation Recovery and T2p-SSFP respectively on a 1.5 T Philips Achieva systems (Philips Healthcare, Best, Netherlands)

**Fig. 4:**
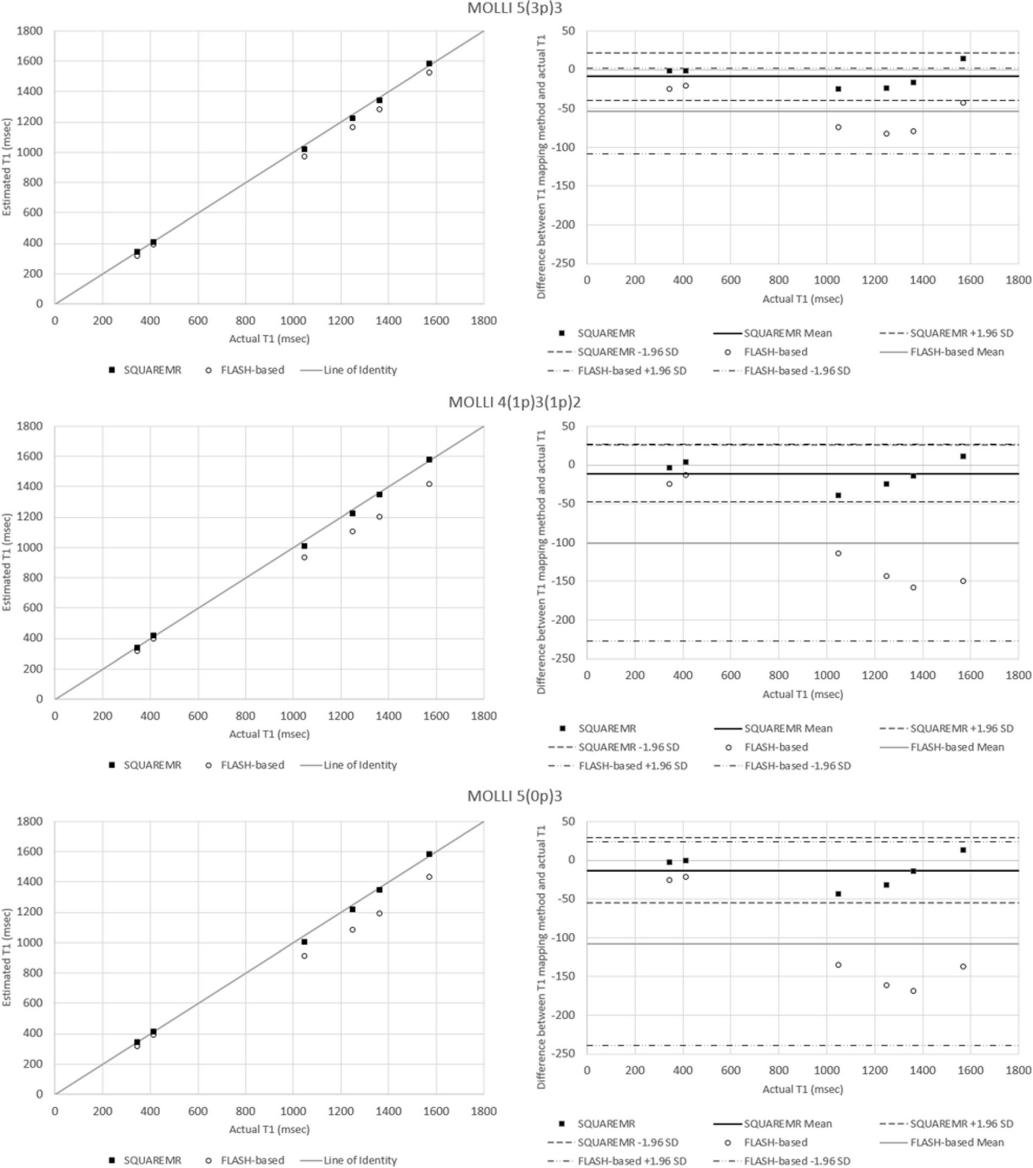
Comparison between SQUAREMR and conventional MOLLI post-processing (FLASH-based) for the myocardial phantom set. Each row of plots represents a separate MOLLI acquisition scheme. The left column shows the deviation of the measured T1 values of the phantoms with SQUAREMR (black *rectangles*) and with conventional (i.e. FLASH-based Look Locker correction factor) MOLLI post-processing (*open circles*) from the line of identity (*gray line*). The right column shows the corresponding modified Bland Altman plots for both methods (solid horizontal lines represent the means, dashed horizontal lines represent the 95 % confidence limits). Compared to conventional MOLLI post-processing, SQUAREMR presented high accuracy and a similar behavior across the different MOLLI schemes

Results from the first phantom are shown in Fig. 4. For the range of relaxation times corresponding to cardiac tissues (pre- and post- contrast) that were studied, SQUAREMR presented high accuracy and a similar behavior across the different MOLLI schemes. For the first phantom, SQUAREMR demonstrated better accuracy compared to conventional MOLLI post-processing [5(3p)3 scheme bias of 8.8 ± 15.3 msec vs. 53.6 ± 28.2 msec, *p* < 0.05; 4(1p)3(1p)2 scheme bias of 11 ± 18.8 msec vs. 100.2 ± 64.9 msec, *p* < 0.05; 5(0p)3 scheme bias of 13.1 ± 21.1 msec vs. 107.9 ± 66.9 msec, *p* < 0.05; *N* = 6], even in cases of “tissues” with long T1s and MOLLI schemes that do not allow for full relaxation of long T1s prior to the next inversion. Conventional MOLLI post-processing in these cases presented a bias higher than 100 msec for the phantoms with high T1 values.

**Fig. 5:**
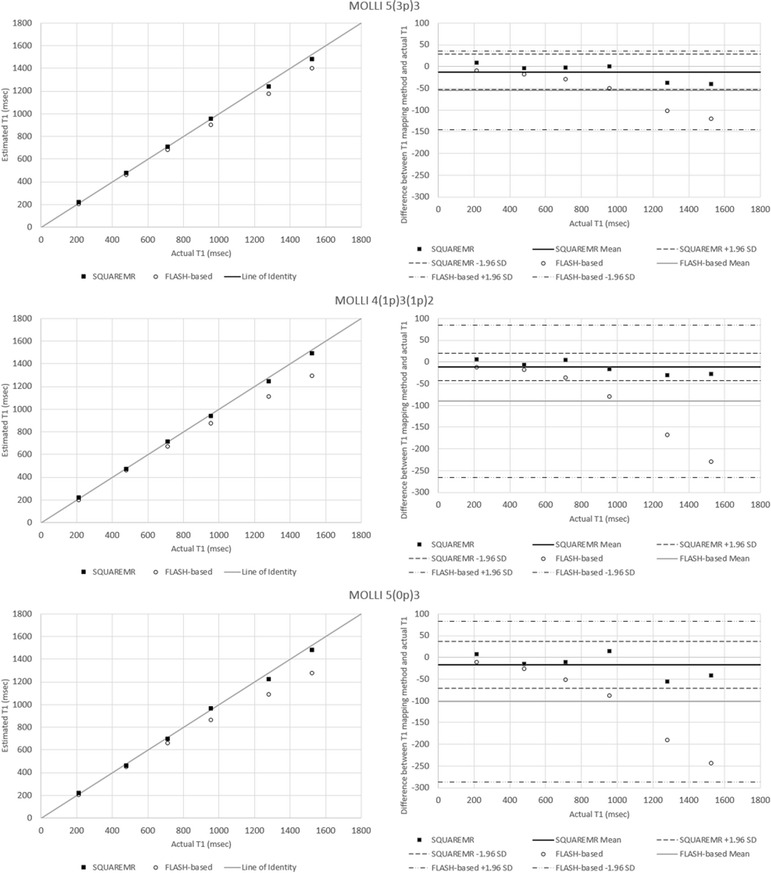
Comparison between SQUAREMR and conventional MOLLI post-processing (FLASH-based) for the Eurospin II phantom set. Each row of plots represents a separate MOLLI acquisition scheme. The left column shows the deviation of the measured T1 values of the phantoms with SQUAREMR (*black rectangles)* and with conventional (i.e. FLASH-based Look Locker correction factor) MOLLI post-processing (*open circles*) from the line of identity (*gray line*). The right column shows the corresponding modified Bland Altman plots for both methods (solid horizontal lines represent the means, dashed horizontal lines represent the 95 % confidence limits). Compared to conventional MOLLI post-processing, SQUAREMR presented high accuracy and a similar behavior across the different MOLLI schemes

In the second phantom, which had close to normal myocardium T2s and a range of T1s, SQUAREMR demonstrated better accuracy than conventional MOLLI [5(3p)3 scheme bias of 12.1 ± 20.5 msec vs. 54.7 ± 45.7 msec, *p* < 0.05; 4(1p)3(1p)2 scheme bias of 11.4 ± 16.1 msec vs. 90.2 ± 89.3 msec, *p* < 0.05; 5(0p)3 scheme bias of 16.9 ± 27.2 msec vs. 101.3 ± 94.3 msec, *p* < 0.05; *N* = 6], (Fig. 5). Conventional MOLLI post-processing introduced an increasing error with increasing T1, as has been previously shown in simulation studies [10].

**Fig. 6:**
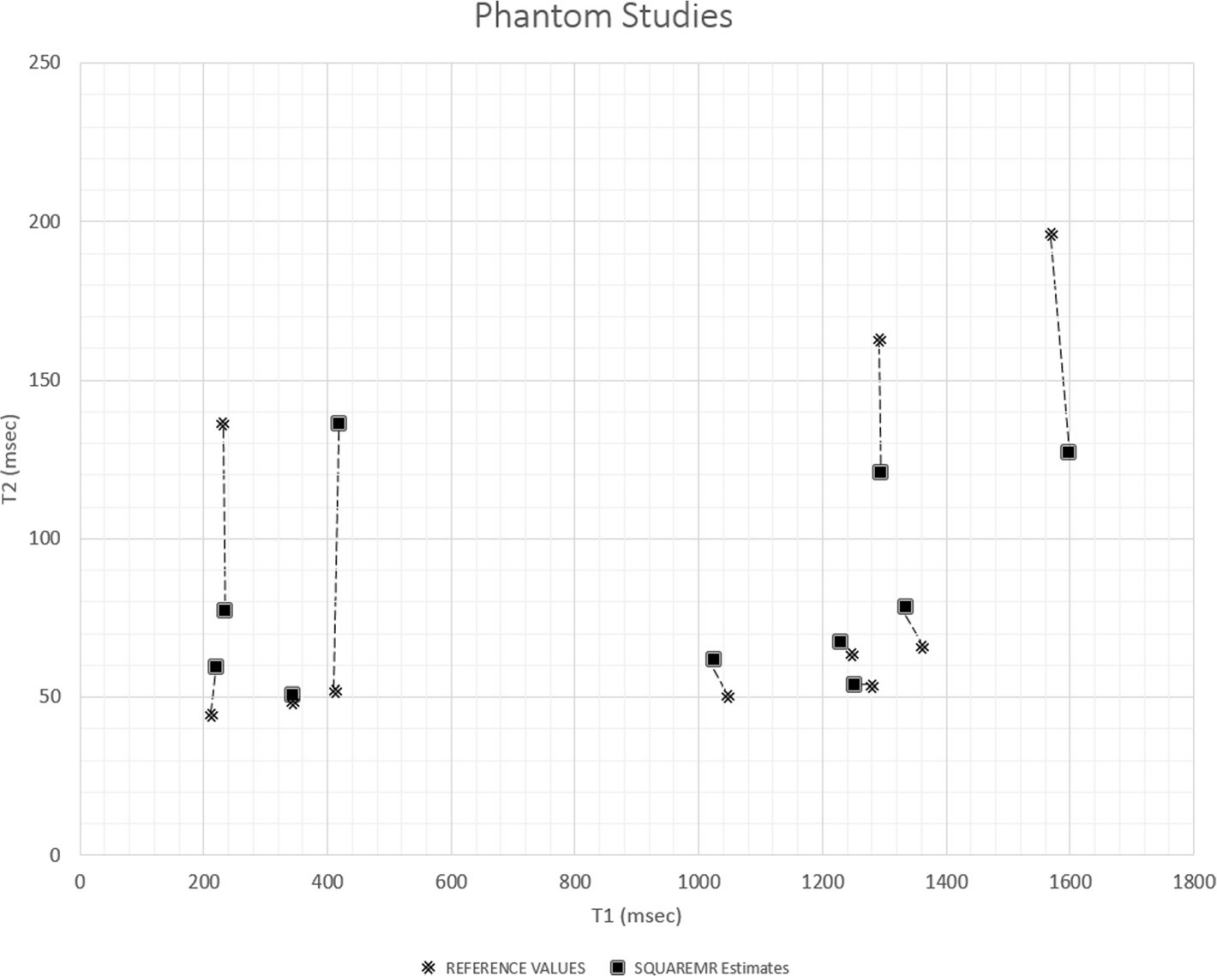
SQUAREMR T1 and T2 estimates of ten phantoms in comparison to their reference T1 and T2 values for a clinically relevant 5(3p)3 MOLLI acquisition scheme. In black rectangles are the T1 and T2 values given by SQUAREMR whereas in black x are the corresponding relaxation constants reference standard values. SQUAREMR demonstrated a small T1 estimation error for all T1 and T2 combinations. The T2 estimation error was small only for phantoms with long T1 and short T2 values. For samples with other T1 and T2 combinations, SQUAREMR demonstrated a larger error in estimating T2 with the MOLLI pulse sequence

Figure 6 shows the SQUAREMR T1 and T2 estimates in phantoms (*N* = 10) against their reference T1 and reference T2 values. SQUAREMR demonstrated small error (14.3 ± 11.2 msec; *N* = 10) in estimating T1 for all T1 and T2 combinations. The error was also relatively small in estimating T2 (7.2 ± 6 msec; *N* = 4) for phantoms with high T1 and low T2 values. For other T1 and T2 combinations, SQUAREMR yielded larger errors (45.3 ± 31.8 msec; *N* = 6) in estimating T2.

### Human studies

**Fig. 7:**
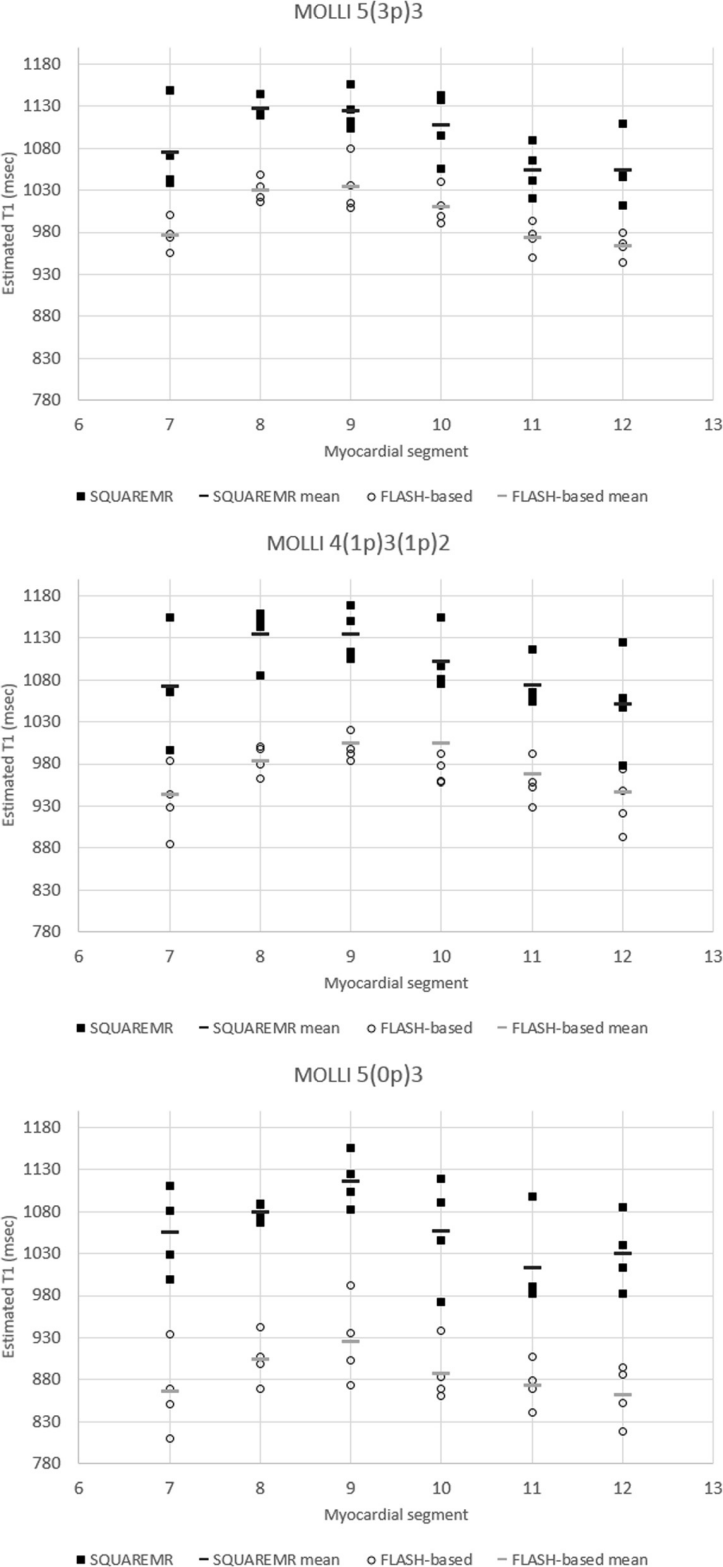
Myocardial T1 values measured from the six myocardial segments (7 to 12). Myocardial T1 values were measured in the mid-ventricular short-axis slices for three different MOLLI acquisition schemes. In black rectangles are the T1 values given by SQUAREMR whereas in black circles are the corresponding T1 values given by the conventional MOLLI post-processing (FLASH-based). In solid black and gray short lines are the mean segmental T1 values for SQUAREMR and conventional MOLLI post-processing respectively. Data were extracted from the four volunteers that were examined additionally with 4(1p)3(1p)2 and 5(0p)3

Figure 7 shows the segmental T1 values given by SQUAREMR and the conventional MOLLI post-processing for the three different MOLLI schemes. SQUAREMR yielded higher T1 values for all segments compared to the conventional MOLLI post-processing, for all three MOLLI schemes. With the MOLLI scheme 5(3p)3, which is being used clinically for pre-contrast myocardial T1 mapping, the average T1 value per short axis slice was 1025 ± 22.9 msec for conventional MOLLI post-processing and 1117 ± 25.6 msec for SQUAREMR (*p* < 0.001, *N* = 12). The average myocardial T1 value for segment 9 (inferior septal myocardium) was 1053 ± 33 msec for conventional MOLLI post-processing and 1148 ± 38 msec for SQUAREMR (*p* < 0.001, *N* = 12). The average blood T1 values were 1570 ± 52.8 msec and 1634 ± 50 msec respectively (*p* < 0.001, *N* = 12). As other studies have already shown [6, 14], a regional variation of segmental average T1 values was measured whereas the average T1 value of myocardial segment 9 was higher compared to the average T1 value of the entire slice.

**Fig. 8:**
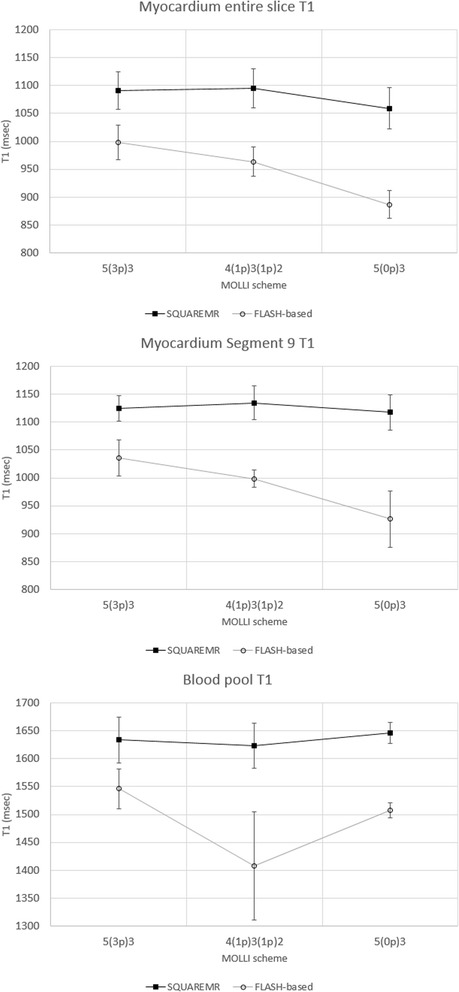
Mean T1 values per short axis slice, myocardial segment nine and blood pool. The mean T1 values were measured with SQUAREMR and conventional MOLLI post-processing for three different MOLLI acquisition schemes. In black rectangles are the mean T1 values given by SQUAREMR whereas in black circles are the corresponding mean T1 values given by the conventional MOLLI post-processing (FLASH-based). Error bars depict the standard deviation of the mean for each MOLLI acquisition scheme. Data were extracted from the four volunteers that were examined additionally with 4(1p)3(1p)2 and 5(0p)3

**Fig. 9:**
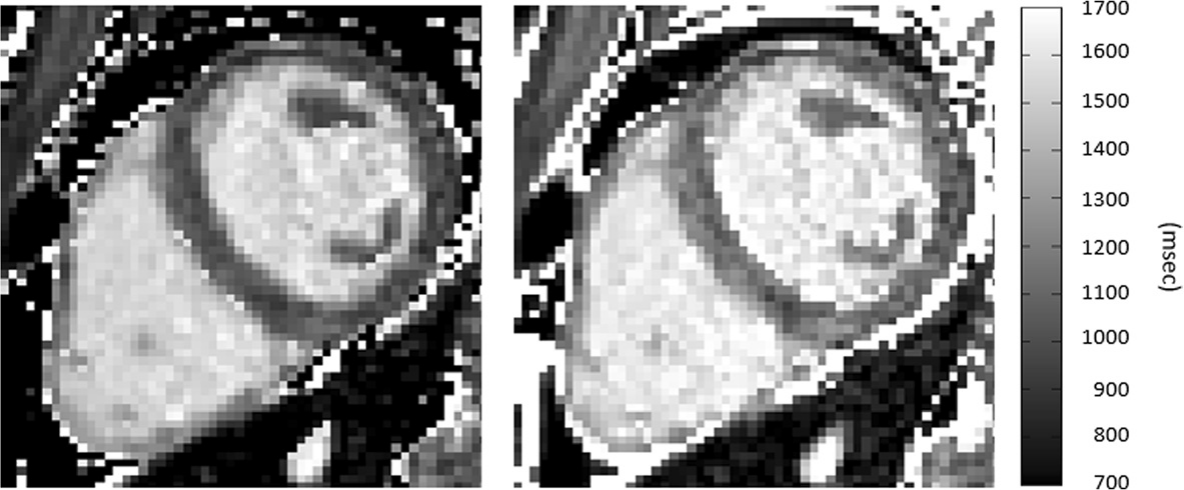
T1 maps of a healthy volunteer. Left image: Conventional MOLLI map derived from a clinical pulse sequence. Right image: T1 map obtained from MOLLI-based tissue signal intensities using SQUAREMR. (MOLLI acquisition scheme 5(3p)3, rBW = 200 kHz, FOV = 272 mm×272 mm, acquisition matrix = 124×124, SENSE acceleration factor = 2). Grayscale images are presented to avoid exaggerating contrast when crossing different color boundaries, which commonly occurs with color images

Figure 8 shows how the T1 values, resulting from SQUAREMR and from the conventional MOLLI post processing, have been affected by the three different MOLLI schemes. The T1 values were measured over the entire slice, myocardial segment 9 and blood pool. SQUAREMR was well behaved with consistent mean T1 values and consistent variance across the different MOLLI schemes. The conventional MOLLI post-processing overall showed significantly different mean T1 values between schemes [e.g. *p* < 0.001 for myocardial 5(3p)3 vs. 5(0p)3]. Table 2 gives the results presented in Fig. 8 whereas Fig. 9 shows T1 maps from a healthy volunteer derived from the conventional MOLLI post processing (left image) and SQUAREMR (right image) for a 5(3p)3 acquisition scheme.

**Table 2:** Mean T1 values (msec) per slice, myocardial segment 9 and blood pool

	Myocardium entire slice T1				Myocardium segment 9 T1				Blood pool T1			
	MOLLI		SQUAREMR		MOLLI		SQUAREMR		MOLLI		SQUAREMR	
MOLLI scheme	Mean	SD	Mean	SD	Mean	SD	Mean	SD	Mean	SD	Mean	SD
5(3p)3	998	31	1091	34	1035	32	1125	23	1546	36	1633	41
4(1p)3(1p)2	964	26	1095	35	998	16	1135	30	1408	97	1623	40
5(0p)3	887	25	1059	37	926	51	1117	31	1507	13	1646	19

T1 values were measured with SQUAREMR and conventional MOLLI post-processing for three different MOLLI acquisition schemes. Data were extracted from the four volunteers that were examined additionally with 4(1p)3(1p)2 and 5(0p)3

### SQUAREMR performance

To illustrate the performance of SQUAREMR, two different sets of pre-Gd MOLLI experiments were examined. The simulation execution times and the database search times for different MOLLI experiment sizes were recorded and are shown in Table 3. It can be seen that the duration of the SQUAREMR processing varied depending on the complexity of the experiment, which can be defined by a number of parameters such as the size of the acquisition matrix, the number of database entries, MOLLI pulse sequence timesteps, etc. The total execution times range from 33 s to 47 min on a server with four Tesla C2075 GPU cards.

**Table 3:** SQUAREMR performance for MOLLI experiments with varying complexity

#	Simulation timesteps	Simulation temporal step (μsec)	Database entries	Sampling step (msec)	T1 map size	Spins along slice	Simulation execution (min:sec)	Database search (min:sec)	Total execution time (min:sec)
1	63705	10	533400	1	128x128	101	17 m:47 s	0 m:29 s	18 m:16 s
2	63705	10	21560	5	128x128	21	0 m:9 s	0 m:24 s	0 m:33 s
3	156877	5	533400	1	320x320	101	44 m:27 s	2 m:47 s	47 m:14 s
4	156877	5	21560	5	320x320	21	0 m:22 s	1 m:50s	2 m:12 s

The simulation execution times and the database search times were recorded for the application of SQUAREMR on MOLLI experiment of varying size

## Discussion

A new method for improving measurements from clinical pulse sequences in CMR relaxometry was presented. The use of parallel simulations was shown to improve the T1 estimates in MOLLI by comparing the MRI signals acquired from the MRI scanner to the entire pool of physiological simulated signals that were produced by parallel simulations of the identical pulse sequence on a population of spins. While the current study explored the feasibility of obtaining T1 properties from MOLLI images, in principle it could be extended in other areas of quantitative MR.

MOLLI T1 mapping is widely used today; however the correction of the T1 recovery relies on the a FLASH closed form expression [5] only because such an expression does not exist for the actual bSSFP readout that the MOLLI pulse sequence utilizes. Several studies have already shown that MOLLI T1 mapping underestimates true T1 whereas its measurement error is influenced by several acquisition protocol parameters [5, 6, 10, 12, 16]. SQUAREMR does not rely on closed form expressions but rather on an extended simulation of the pulse sequence itself. The basic concept of SQUAREMR was based on the premise that realistic simulations of clinical pulse sequences on tissue models of specific parameters (T1, T2) would result in identical signals to the signals acquired from the MRI scanner after the application of the same pulse sequences on true tissues with the same relaxation properties.

In the current work, a CMR protocol consisting of three different MOLLI pulse sequences was implemented: 5(3p)3, 4(1p)3(1p)2 and 5(0p)3. For every MOLLI experiment, the identical MOLLI pulse sequence was simulated for T1 and T2 ranges of 200 to 1900 msec and of 20 to 400 msec respectively taking into account realistic aspects of the MR experiment, such as realistic excitation slice profiles and heart rate variation in in-vivo studies. For that purpose, MRISIMUL, a GPU-based, MR physics simulator [19, 20] was utilized in this study.

The CMR T1 mapping protocol was applied on three phantom setups and on twelve healthy volunteers. All twelve volunteers and phantom setups were examined with the clinical 5(3p)3 scheme. The actual TI timings were extracted from the scanner data and used for the SQUAREMR simulations allowing for more realistic simulations. Four out of twelve volunteers and two out of three phantom setups were imaged additionally with 4(1p)3(1p)2 and 5(0p)3. Results of the CMR protocol on the phantom setups demonstrated improvement of accuracy and a substantially reduced T1 variability across the different MOLLI schemes. Compared to conventional MOLLI post-processing, SQUAREMR showed improved accuracy even for long T1s with no pause between modified-Look-Locker experiments. The latter demonstrates that the T1 estimates are not dependent on the duration of the delay between successive modified Look-Locker experiments, which in patient scans may change due to heartrate variations.

In order to investigate whether SQUAREMR could also provide T2 estimates from data obtained with a MOLLI pulse sequence, the methodology was applied on phantoms with a range of T1 and T2 values. SQUAREMR showed reasonable T2 values in phantoms with long T1s and short T2s. Larger errors in estimating T2 were observed for the remaining T1 and T2 combinations; however, this was expected for a T1 mapping pulse sequence, such as MOLLI. Previous studies have shown that the MOLLI pulse sequence introduces some T2 modulation on the signal, which is more pronounced for long T1 and short T2 values [10, 34]; therefore, for these values SQUAREMR provided reasonable T2 estimates. For other T1s and T2s, SQUAREMR was unable to provide reasonable T2 estimates since the MOLLI signal simply did not contain this information. The T2 results shown in Fig. 6 indicate that this was not a limitation of SQUAREMR in terms of convergence but rather a limitation imposed by the MOLLI pulse sequence itself which has been designed to mainly modulate the MOLLI signal based on the T1 values of the tissues irrespective of their T2 values.

In in-vivo studies, myocardial and blood T1 values were measured. The average T1 values obtained with the CMR protocol for both the conventional MOLLI post-processing and SQUAREMR are summarized in Table 2 for *N* = 4. SQUAREMR showed substantially elevated T1 values for both myocardium and blood pool when compared to conventional MOLLI post-processing. Conventional MOLLI T1 values in this work were similar to values previously reported in the literature. In particular, the conventional MOLLI scheme 5(3p)3 yielded an average blood T1 value of 1570 ± 52 msec (*N* = 12). Previous studies have reported 1534 msec with MOLLI [27] and 1516 ± 21 msec [13] with saturation recovery FLASH. Also, in this work, the conventional MOLLI T1 value for myocardium (segment 9) of 1053 ± 33 msec (*N* = 12) was similar to that of previous studies with MOLLI (1034 ± 56 msec [35] and 1052 ± 41 msec [11]). On the other hand, SQUAREMR resulted in MOLLI T1 values similar to more accurate CMR T1 mapping techniques. In particular, the average blood T1 value of 1634 ± 50 msec (*N* = 12) was close to that previously reported with SASHA (1639 ± 97 msec [3]). The average T1 value in myocardial segment nine obtained with SQUAREMR MOLLI was 1148 ± 38 msec (*N* = 12) which was close to that previously reported with rapid cardiac gated single-shot IR-FSE sequence (1092 ± 64 msec [8]) and two dimensional SASHA (1105 ± 46 msec [36]).

The application of a parallel realistic simulator in order to correct measured data from the scanner is a new concept. In the past, MR simulations have been used in a limited scope [10, 16, 17] with the exception of MR-fingerprinting [15]. SQUAREMR depends on extended and realistic MR simulations of already available clinical pulse sequences in order to build an extensive database of simulated signals identical (ideally) to the signals obtained from the MR scanner for the same experiment configuration. The utilization of multi-GPU technology along with the technology advancements taking place on the GPU hardware suggest that this method has the potential to become a real-time routine on the MRI scanner in the future.

In this work, some limitations apply. Simulation of magnetization transfer (MT) was not studied although previous simulation studies [10, 37] suggest that MT plays a role in T1 underestimation with MOLLI. However, accurate simulation of MT becomes challenging for the entire range of relaxation times being studied in this work since previous work has shown alteration of MT in disease cases (e.g. myocardial infarction [38]) and among different tissue types (blood vs. myocardium) [10]. Also, blood flow effects were not studied in this work. Simulations assumed a stationary spin model during the application of MOLLI pulse sequence. Although the bSSFP readout is applied during diastasis, when the heart muscle is mostly stationary, blood flow effects may alter the apparent T1 relaxation in the blood pool and, in turn, the T1 estimation [10]. In this implementation, a linear full search of the database was utilized, which limited the performance of SQUAREMR in terms of its execution speed. Non-linear optimization might be way forward but were not tested. In the future, optimization of algorithms for database construction (such as using a variable step for T1 and T2 values, eliminating database T1, T2 pairs not pertaining to a particular MR application, etc.) and database search (such as using data specific schemes) could further improve overall SQUAREMR performance. Despite these limitations, SQUAREMR demonstrated improved T1 accuracy in phantom studies whereas in healthy volunteer studies the reported T1 values of myocardium and blood were close to those acquired with more accurate CMR T1 mapping techniques in the literature. Last but not least, the substantially reduced T1 variability across the different MOLLI schemes with SQUAREMR suggests that myocardial tissue characterization could potentially be achieved within 8 heart beats.

## Conclusion

In conclusion, SQUAREMR is a new method that allows for quantification of CMR data with already available clinical pulse sequences and with the aid of comprehensive, parallel MRI simulations. In this study, a MOLLI-based T1 mapping example was investigated demonstrating improvement of accuracy in phantom studies and consistent mean T1 values and consistent variance across the different MOLLI schemes in humans. This was true even for a wide range of T1 values with MOLLI schemes with no pause between modified-Look-Locker experiments, indicating potential value for myocardial tissue characterization within just 8 heart beats.

The methods presented in this study represent a different approach in quantitative CMR with existing clinical pulse sequences. SQUAREMR allows for correction of quantitative CMR data (e.g. MOLLI T1 maps) that have already been acquired by simulating the clinical pulse sequence that was used for the data acquisition. Last, it is expected that in the future SQUAREMR may improve data consistency and advance quantitative MR to become more robust across imaging centers, vendors and experimental configurations and the technique may be extended in other areas of quantitative MR imaging.

### Ethics, consent and permissions

The study was approved by the local ethics committee and all subjects provided written consent for publication of this study and accompanying images (The regional ethics committee, Lund, Sweden. Ethics application number: 541/2004).
